# Protein purification strategies must consider downstream applications and individual biological characteristics

**DOI:** 10.1186/s12934-022-01778-5

**Published:** 2022-04-07

**Authors:** Kim Remans, Mario Lebendiker, Celeste Abreu, Mariano Maffei, Shaila Sellathurai, Marina M. May, Ondřej Vaněk, Ario de Marco

**Affiliations:** 1 European Molecular Biology Laboratory (EMBL), Meyerhofstrasse 1, 69117 Heidelberg, Germany; 2 Protein Purification Facility, The Wolfson Centre for Applied Structural Biology, The Hebrew University of Jerusalem, 91904 Jerusalem, Israel; 3 Department of Biochemistry, Faculty of Science, Charles University, Hlavova 2030/8, 12840 Prague, Czech Republic; 4 Evvivax Biotech, Via di Castel Romano 100, 00128 Rome, Italy; 5 Takis Biotech, Via di Castel Romano, 100, 00128 Rome, Italy; 6 AiCuris Anti-Infective Cures AG, Friedrich-Ebert-Str. 475, 42117 Wuppertal, Germany; 7 Lab of Environmental and Life Sciences, University of Nova Gorica, Vipavska Cesta 13, 5000 Rožna Dolina-Nova Gorica, Slovenia

**Keywords:** Recombinant proteins, Protein quality control, Protein functionality, Purification strategies

## Abstract

**Background:**

Proteins are used as reagents in a broad range of scientific fields. The reliability and reproducibility of experimental data will largely depend on the quality of the (recombinant) proteins and, consequently, these should undergo thorough structural and functional controls. Depending on the downstream application and the biochemical characteristics of the protein, different sets of specific features will need to be checked.

**Results:**

A number of examples, representative of recurrent issues and previously published strategies, has been reported that illustrate real cases of recombinant protein production in which careful strategy design at the start of the project combined with quality controls throughout the production process was imperative to obtain high-quality samples compatible with the planned downstream applications. Some proteins possess intrinsic properties (e.g., prone to aggregation, rich in cysteines, or a high affinity for nucleic acids) that require certain precautions during the expression and purification process. For other proteins, the downstream application might demand specific conditions, such as for proteins intended for animal use that need to be endotoxin-free.

**Conclusions:**

This review has been designed to act as a practical reference list for researchers who wish to produce and evaluate recombinant proteins with certain specific requirements or that need particular care for their preparation and storage.

**Supplementary Information:**

The online version contains supplementary material available at 10.1186/s12934-022-01778-5.

## Introduction

Upstream recombinant protein processing in the industry is a highly-regulated practice performed following precise standard operating procedures. A similar consistency (in procedures) is difficult to observe at the academic level, primarily due to the misleading assumption that such a widely used technology does not require specific expertise and training to obtain usable proteins. The consequence of disregarding good practices and ignoring the necessity of checking the quality of the final products has been recently analyzed and prompted the publication of guidelines for the evaluation of purified recombinant proteins [1, 2] (for updates, see https://p4eu.org/protein-quality-standard-pqs and https://arbre-mobieu.eu/guidelines-on-protein-quality-control). The purpose of these guidelines is to improve the reproducibility of data obtained with protein reagents to make the whole process more transparent and encourage the use of good practices for protein preparation, data analysis, presentation, and reporting. Since the implementation of these recommendations requires not only their reliability but also their feasibility, the proposed guidelines focus on a small number of basic analyses, such as gel filtration and SDS-PAGE, that are easy to perform with widely available instrumentation.

**Fig. 1:**
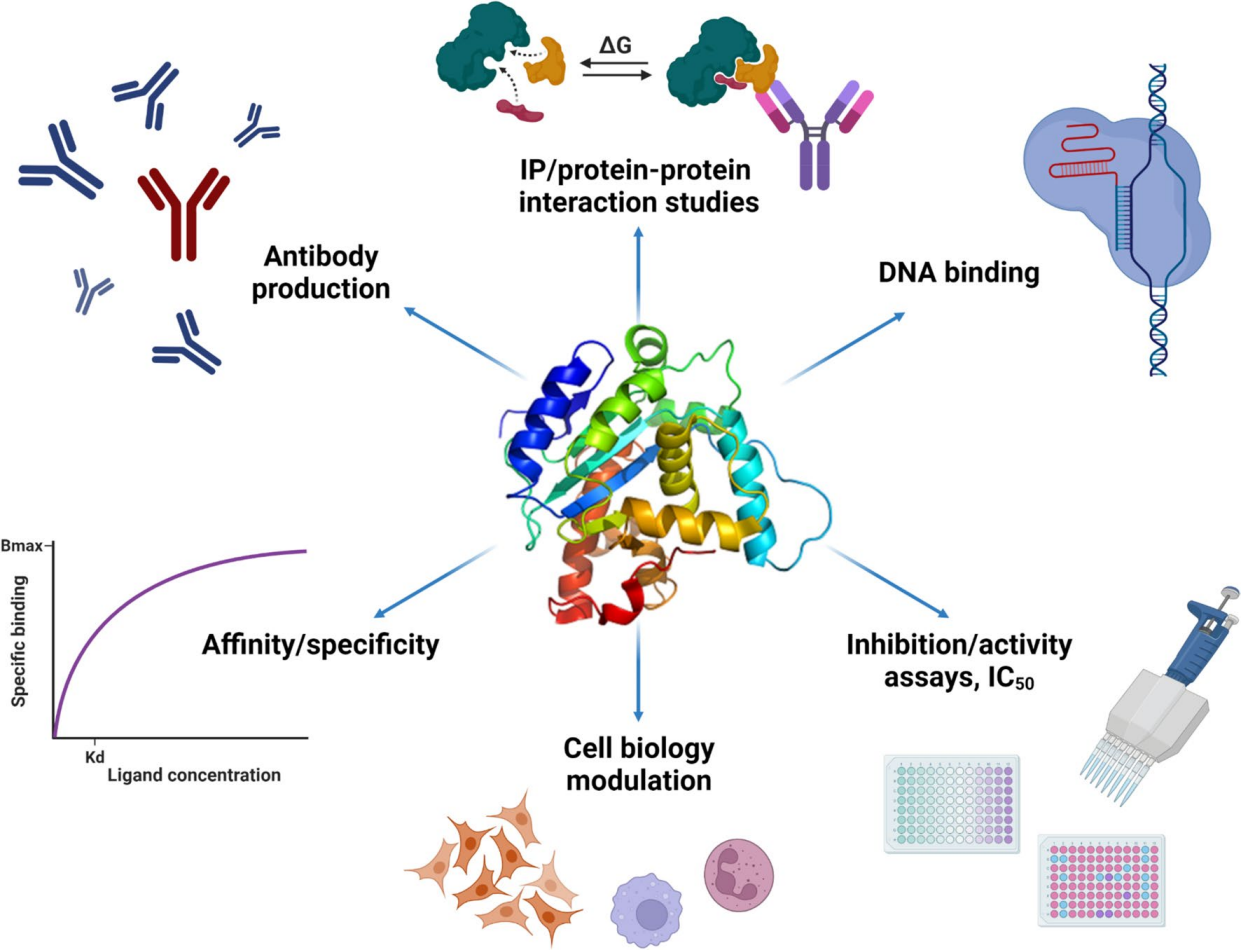
Recombinant proteins: useful reagents for many different applications. Proteins can function as the object of scientific research but can also be used as reagents and tool molecules. For example, mechanistic insights into protein function can be obtained by elucidating the 3D structure and studying interactions with other proteins, nucleic acids, or small molecules by determining affinities and specificities. Antibodies can be helpful tools to identify targets, whereas proteins such as cytokines and growth factors can be used as reagents in cell biology assays

The present review aims to relate the original indications/recommendations, that are generally useful and can be applied to any recombinant protein, to proteins with specific biochemical characteristics or the use of which needs particular requirements (Fig. 1). Such proteins (prone to aggregate, possessing disulfide bonds, stabilized by divalent cations, etc.) or proteins produced for particular biological applications require ad hoc assessment to achieve full functionality and enable high-quality downstream results (and avoid time-consuming troubleshooting). For each end-use, different quantities and levels of sample purity are required and this implies alternative purification strategies to avoid, for instance, the presence of endotoxins or other toxic components when the protein is used in cell assays or injected into animals, or of contaminant nucleic acids in proteins assessed for their interaction with such macromolecules. In Table 1, an overview can be found of the specific requirements and possible strategies for proteins intended for use in certain biological applications or with particular biochemical properties.

**Table 1:** QC requirements and strategies for proteins that are used in specific biological applications or have particular intrinsic biochemical features

	QC requirements	Strategies
*Biological applications*		
Protein–protein interactions studies (pull-down assays, IP, crosslinking protein interaction analysis, ITC, MST, DLS, and others)	QC Guidelines [1, 2]	Proteins must be properly folded and contain the correct oligomeric conformation to avoid “non-specific” sticking to soluble aggregates
DNA/RNA interaction assays (EMSA, ITC, MST, …)	QC Guidelines [1, 2] Check that nucleases and nucleic acids were removed	Nucleic acid removal step in the purification process: anion exchange, polyethyleneimine, or streptomycin sulfate precipitation Avoid the use of external DNases or RNases
Structural analysis (X-ray crystallography, cryo-EM, NMR)	Folded, stable protein as suggested in the QC Guidelines [1, 2] Suitable storage buffers	Conditions must be determined in which the protein remains correctly folded and in the appropriate oligomeric state at the high protein concentrations required for most structural biology applications
Antibody fragment production	QC Guidelines [1, 2] Activity evaluation	Affinity measurement, IP, ELISA
Studies with sensitive cell lines, in vivo experiments, and production of growth factors for cell culture	QC Guidelines [1, 2] Endotoxin-free Confirmed activity	Endotoxin removal via ion exchange, SEC, and/or endotoxin removal beads. Endotoxin removal by chromatographic washing steps with non-ionic detergents such as Triton X114
Protein production for in vivo assays	QC Guidelines [1, 2] with emphasis on purity Non-toxic buffer for animal use Endotoxin-free	Check buffer compatibility with host animals. Final buffer free of components toxic for the animals (endotoxins)
Antigen production for animal immunization	QC Guidelines [1, 2] with emphasis on purity Non-toxic buffer for animal use Endotoxin-free	Check buffer compatibility with host animals. Final buffer free of components toxic for the animals (endotoxins)
Antigen production for in vitro panning	QC Guidelines [1, 2] with emphasis on purity and monodispersity Native folding	Check aggregation and presence of hydrophobic patches. If feasible, check functionality
Compound screening, Inhibition/activity assay development	QC Guidelines [1, 2] Folded, stable protein	Buffer compatibility Absence of contaminating inhibitors, interferents, etc
Affinity/specificity measurements	Folded, stable protein QC Guidelines [1, 2]	Buffer compatibility Absence of contaminating inhibitors, interferents, etc
*Intrinsic biochemical features*		
Protein complexes	QC Guidelines [1, 2] with emphasis on a correct protein stoichiometry by SDS-PAGE and correct mass by SEC-MALS or similar methods	Careful strategy design. Optimization of expression conditions Use mild conditions that avoid complex dissociation during all the purification procedures. Recovery of the complete and stable protein complex in the correct oligomerization state and correct protein stoichiometry
Prone-to-aggregation proteins	QC Guidelines [1, 2] A repeat of the QC checks after protein storage (before usage) is highly recommended	Optimization of expression conditions Optimization of the buffer and/or storage conditions with the help of techniques such as thermofluor, nano-DSF, DLS Rapid strategy for purifying and storing the target protein as fast as possible Work at low temperature and avoid reaching critical protein concentrations that induce aggregation
Proteins binding to divalent cations or other co-factors	QC Guidelines [1, 2] Optimally, verification of divalent cation/co-factor incorporation via spectroscopy	Addition of the divalent cation or co-factor to the growth medium and/or the purification buffers. Recovery of properly folded protein with the divalent cation (or another co-factor) correctly incorporated. Avoid the use of chelating agents in the buffers
Proteins with inter- or intramolecular disulfide bonds	QC Guidelines [1, 2] SDS-PAGE with sample buffer with or without reducing agents Assess correct disulfide bond formation by DTNB (Ellman’s reagent) or MS	Optimization of expression conditions Avoid the addition of reducing agents for the recovery of stable protein with native disulfide bonds
Proteins with free cysteines	QC Guidelines [1, 2] Assess undesirable disulfide bond formation by MS	Avoid undesirable disulfide bond formation by using reducing agents during purification and storage

We recommend always performing buffer optimization, both for protein purification and storage. Techniques that can be used for this are, for example, thermofluor stability assays, nano-DSF, nano-DLS, etc. The quality of the final purified protein should be evaluated according to the following QC Guidelines* [1, 2]:

•Purity by SDS-PAGE, capillary electrophoresis (CE), and others

•Homogeneity/dispersity by dynamic light scattering (DLS), size-exclusion chromatography (SEC), or, preferably, by SEC coupled to multi-angle light scattering

•Identity and integrity by either ‘bottom-up’ MS (mass fingerprinting or tryptic digests) or ‘top-down’ MS (measuring intact protein mass)

On the top of these universal tests, specific QC analyses might be necessary and the most common examples of demanding samples are reported below, together with indications about how to reach satisfactory results. Such indications are derived from the authors’ professional experience (see the cases reported in the main text) and are not totally exhaustive but rather represent options to address the listed issues

This article will describe a set of real-life examples to illustrate the most important issues to consider during the expression and purification process of such proteins and the strategies to overcome the most common pitfalls.

### Demanding protein production—Examples of problem identification, mitigation strategy design, and corresponding output

#### Nucleic acid-binding proteins

For proteins that interact with nucleic acids, it is essential to introduce a nucleic acid removal step at some point in the purification workflow (Table 1). During the cell lysis process, nucleases such as *Sm* nuclease (benzonase®) and/or DNase or RNase can be added. However, this is often not sufficient to get rid of protein-bound nucleic acids and is also not recommended in all cases, as this could lead to the requirement to remove both the added nucleases and the contaminating nucleic acids. Therefore, extra or alternative nucleic acid removal steps such as a polyethyleneimine (PEI) or streptomycin sulfate precipitation, a heparin purification or an ion exchange chromatography step are usually required to obtain nucleic acid-free protein (Additional file 1: Examples S1 and S2). The presence of nucleic acids can be monitored throughout the purification process via the A_260nm_/A_280nm_ ratio. If this ratio is below 0.6, it is a good indication of pure protein with minimal nucleic acid contamination. For specific cases of particularly strong nucleic acid binding, a more stringent procedure using a denaturant such as urea can be used, followed by protein refolding. Care should be taken to use good quality reagents, fresh sterile buffers, and clean columns (washed with 0.5 M NaOH before use).

#### Mouse Ferritin heavy chain 1

**Fig. 2:**
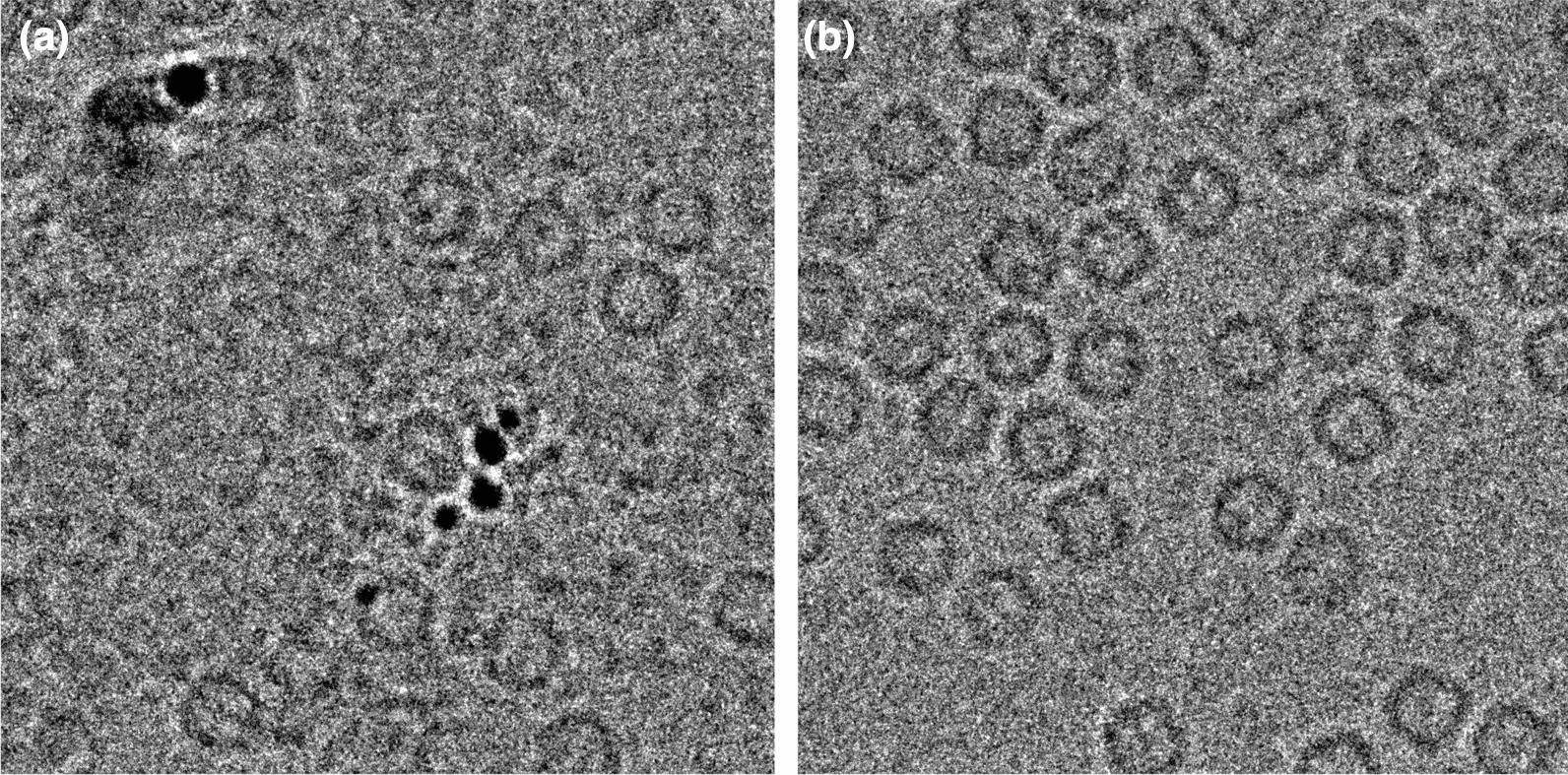
Mouse Ferritin heavy chain 1. **a** Cryo-EM image of mouse Ferritin heavy chain 1 (mFth1) before optimizing the purification process. The image clearly shows the presence of various contaminants. **b** Cryo-EM image of mFth1 after optimization of the purification process, resulting in pure protein without the presence of contaminants. (Original figure from Remans’ lab)

The goal of producing purified mouse Ferritin heavy chain 1 (mFth1) protein was to use it for high-resolution single-particle cryogenic electron microscopy (cryo-EM). The *E. coli* expression construct encoded untagged mFth1, and the initial protocol described cell lysis by sonication without adding any nucleases and a heating step at 70 °C followed by an ammonium sulfate precipitation, dialysis, and a size-exclusion chromatography step (work performed at Remans’ lab, according to the initial protocol published by Danev et al*.*, 2021 [3]). However, this sample showed the presence of large amounts of contaminants in the cryo-EM images (Fig. 2A), making it wholly unsuitable for its purpose. To optimize the protocol and increase purity, some extra steps were introduced in the purification workflow and the presence of contaminating nucleic acids was monitored via the A_260nm_/A_280nm_ ratio. Firstly, *Sm* nuclease was added both before the cell lysis process and during dialysis after the ammonium sulfate precipitation step. As the A_260nm_/A_280nm_ ratio was still too high, the sample was subsequently subjected to an anion exchange chromatography step (HiTrap Q HP), leading to a reduction of the A_260nm_/A_280nm_ ratio from 1.4 to 0.8. After the size-exclusion chromatography (HiLoad® 16/600 Superdex® 200 pg), the A_260nm_/A_280nm_ ratio of the final mFth1 sample was 0.56, and the protein looked very pure on SDS-PAGE, indicating a proper removal of the contaminants, which was also confirmed in the cryo-EM images (Fig. 2B).

#### The chimeric protein human dsRBEC (dsRBD-EGF-chimera)

dsRBEC comprises the dsRNA binding domain (dsRBD) of human Protein Kinase R (hPKR), which serves as the polyIC (polyinosinic/polycytidylic acid) binding moiety, and it is fused to human EGF (hEGF), the targeting moiety. dsRBD shows an extremely high binding capacity for dsRNA when expressed in *E. coli* and such contaminants must be completely removed before polyIC addition and selective delivery into EGFR over-expressing tumor cells.

After bacterial cell lysis, most of the recombinant protein is insoluble, and the contaminant RNA impairs binding of the recombinant protein present in the soluble fraction from binding to the first capture IMAC column. Conventional procedures like polyethyleneimine (PEI) or streptomycin sulfate precipitation, RNase treatment, or high salt buffer failed to eliminate the RNA. However, the addition of urea (4 M) allowed a complete release of the contaminant, as can be clearly detected in the figures reported in Additional file 1: Fig. 1 [4]. Therefore, the refolding conditions were optimized to find the most suitable buffers and additives. Finally, cell lysis was performed in buffer supplemented with 4 M urea and the supernatant was loaded on an IMAC column, followed by a slow overnight refolding gradient from 4 to 0 M urea in optimized refolding buffer (on-column refolding). The dsRBEC was eluted from the IMAC column with refolding buffer plus imidazole and then immediately applied to a preparative Superdex 75 (100 × 1.6 cm in tandem with 60 × 1.6 cm ~ 320 ml) gel filtration for final polishing [4, 5]. The resulting dsRBEC, charged with polyIC, effectively and selectively induced polyIC internalization into EGFR overexpressing cells, inducing cell death and cytokine secretion.

#### Proteins that bind to divalent cations or other co-factors

For proteins that interact with divalent cations such as Zn^2+^, Fe^2+^, Cu^2+^, or other co-factors, the addition of a specific divalent cation (or other co-factor) during the protein expression process can be crucial (Table 1). The presence of low quantities of the same divalent cations (or other co-factors) might be necessary during the purification workflow as well. In the case of divalent cations, it is imperative to avoid using any chelating agent such as EDTA, EGTA or reducing agents with chelating properties such as DTT or DTE. When working with divalent cation-binding proteins, care must be taken to only use chelating-free (commercial) protease inhibitor cocktails. The actual presence of the divalent cations (or other co-factors) in the purified protein should be confirmed spectroscopically when possible, for instance, by atomic absorption spectrophotometry (Additional file 1: Example S5).

#### Recombinant ferredoxin from thermophilic cyanobacterium *Mastigocladus laminosus* (rFd) containing a single [2Fe ± 2S] cluster

Ferredoxins are soluble iron-sulfur proteins involved in electron-transfer reactions. Plant-type ferredoxins, which carry a single [2Fe ± 2S] cluster, serve as electron acceptors of Photosystem I. The recombinant rFd protein was expressed in *E. coli* strain BL21 (DE3) (Novagen) grown in TB medium containing 10 mM FeCl_3_ and antibiotics. The elution from the IMAC capture column was performed with a buffer containing 10 mM histidine instead of imidazole since imidazole has been shown to disrupt the [2Fe ± 2S] cluster [6]. Further purification steps included an anion exchange and size-exclusion chromatography. Finally, the rFd protein was concentrated to 12 mg/ml for crystallization screening. Interestingly, a higher resolution was obtained when crystallizing the native ferredoxin purified from the cyanobacterium *Mastigocladus laminosus* instead of the recombinantly produced ferredoxin.

#### Proteins used as antigens

Proteins are often exploited as antigens for the recovery of target-specific ligands. The first point to consider is whether the proteins will be used for an in vivo immunization with the aim of obtaining conventional mono- or polyclonal IgG or if antigens will be exploited in a procedure involving camelid IgG and/or an in vitro selection process (Table 1).

#### Antigens used for conventional antibody production

When antigens are injected into mice to finally obtain hybridoma cells and produce monoclonal IgG antibodies, the correct folding of the protein is not crucial. This is because most antibodies will recognize linear epitopes that are not particularly affected by the antigen structure. Even denatured antigens, for instance recovered from inclusion bodies (Additional file 1: Figure S3), can generate a valid immune response ([7], Additional file 1: Example S3). However, in this case, the sample purity must be carefully evaluated because minor contaminations with highly immunogenic proteins can induce a strong response at the expense of the target antigen.

#### Panning of binder libraries

Additional care must be taken when dealing with libraries obtained from camelids immunized to produce collections of binders that underwent somatic maturation. It is known that a large number of camelid antibodies binds to structural epitopes that better fit their convex paratopes. This implies that the antigen used for immunization must conserve the tridimensional native protein conformation to induce the production of antibodies able to recognize the epitopes available in vivo. There is no straightforward explanation of how this selection works because the peptide presentation modality of the class II camelid major histocompatibility complex (MHC) is conserved among mammals [8]. The antigen is digested, and single peptides of usually 13–17 amino acids are then presented on the MHC of professional antigen-presenting cells. Apart from specific cases, in most species this process results in the availability of peptides with a limited tertiary structure that will promote the development of antibodies recognizing mostly linear epitopes. However, empirically it seems that the correct folding of antigens injected in camelids might substantially help in obtaining nanobodies with excellent binding properties [9].

Similarly, entirely in vitro selections obtained by panning large pre-immune (synthetic or naïve) libraries of antibody fragments or alternative ligand scaffolds require antigens with precisely the same structure as the target to be detected in the final application. The panning procedure has no bias, in the sense that the billions of different available clones have, at least theoretically, the same chances to bind to the epitope offering the best docking among the infinite configurations present on the antigen surface. It means that it is statistically possible to select binders towards both native and new spurious epitopes if these exist due to partial misfolding, aggregation, or different conformations (Additional file 1: Figure S4). Therefore, if the final aim is to detect the antigen in natural conditions, the protein used as the antigen for the panning should be in its native structure; otherwise, the procedure high effectiveness will result in the accumulation of binders specific for epitopes present in artifacts (Additional file 1: Example S4). This is particularly important when nanobody libraries are screened because such molecules prefer to bind to conformational epitopes that could be lost or newly formed due to antigen structural rearrangements.

#### Proteins that contain inter- or intramolecular disulfide bonds or free cysteines

Disulfide bonds are critical for stabilizing the native structures of proteins, particularly proteins exposed to oxidizing environments, like secreted proteins or protein domains exposed on the external cell surface. On the other hand, cytoplasmic proteins are mainly present in the reduced (-SH) form. The correct formation of disulfide bridges depends on the redox conditions and on the availability of disulfide isomerases able to supervise the accomplishment of the native cis and trans bonds (Table 1).

Since many (eukaryotic) proteins undergo further complex post-translational modifications as well, eukaryotic systems are often chosen to produce proteins harboring disulfide bonds. Nevertheless, several bacterial expression systems can provide good yields and sufficient quality in the case of proteins with a limited number of cysteines [10, 11], utilizing specialized *E. coli* strains that enable disulfide bond formation in the cytosol (e.g., SHuffle T7 Express, Origami 2(DE3), Rosetta-gami 2(DE3), etc.), co-expression of sulfhydryl oxidase, or periplasmic accumulation.

During the purification process, the addition of reducing agents must be avoided when purifying proteins that contain inter- or intramolecular disulfide bridges to avoid changes in protein conformation that may alter the function of the protein. In contrast, for proteins that contain free cysteines, the presence of reducing agents is essential during all steps of the purification process and, crucially, during storage. This is important to avoid the formation of artefactual disulfide bonds, which can eventually lead to aggregation.

The most commonly used reducing agents are dithiothreitol (DTT), β-mercaptoethanol (β-ME) and tris-(2-carboxyethyl) phosphine hydrochloride (TCEP). TCEP is a non-thiol and odorless compound, stable in aqueous solutions and resistant to air oxidation. Moreover, unlike DTT, TCEP retains its reducing ability both at acidic pH and at pH above 7.5 [12]. For IMAC resins incompatible with DTT or TCEP, it is advisable to use 5–15 mM β-ME, which can then be replaced by other reducing agents in the following steps. Proteins containing a mixture of disulfide bonds and free cysteines represent a problematic intersection. As a compromise, it is recommended to either use no reducing agents at all or to include a very low concentration of β-ME (2 mM).

Disulfide bonds can be experimentally determined or predicted from the amino acid sequence using bioinformatic tools such as the Disulfide Bond Prediction server of Liu’s Lab (http://liulab.csrc.ac.cn:10003/index), DiANNA (http://clavius.bc.edu/~clotelab/DiANNA/), DISULFIND (http://disulfind.disi.unitn.it/), and diSBPred (https://cs.uno.edu/~tamjid/Software.html).

#### Recombinant antibody fragments

**Fig. 3:**
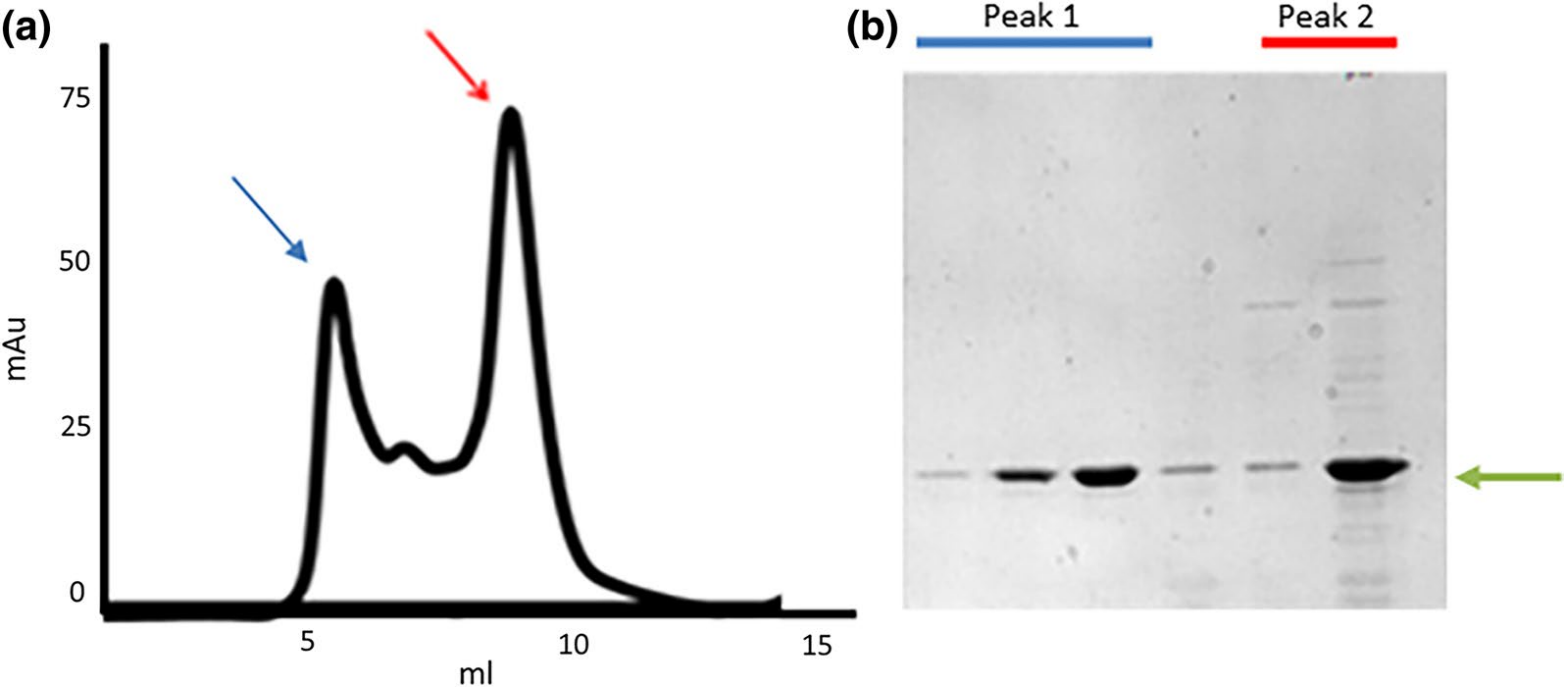
Separation of nanobody soluble aggregates by gel filtration. **a** Gel filtration profile of a nanobody sample. The first peak (blue arrow) corresponds to the void volume, the last (red arrow) to the monomeric molecule. **b** SDS-PAGE loaded with gel filtration elution fractions corresponding to first (blue), intermediate (no color), and last (red) peak. The fractions present different purity degrees, but the nanobody (green arrow) is always the major protein. (Original figure from de Marco’s lab)

In the last years, interest in the production of recombinant antibody fragments has strongly increased. As they are used mainly as reagents, the required quality control checks are prototypic for disulfide bond-dependent proteins. Despite having a simplified structure with respect to IgGs, all antibody-derived fragments possess one or more disulfide bonds. There are several approaches for favoring the correct formations of such bonds, even in bacteria [11]. However, the yields of native, functional reagents are very case-dependent, with the accumulation of misfolded or partially folded proteins in both soluble and insoluble fractions. Furthermore, even correctly folded antibody fragments such as nanobodies might have the propensity to form soluble aggregates (colloidal aggregation) due to non-specific interactions mediated by surface patches [13]. Although removing insoluble fractions is straightforward, soluble aggregates are more challenging to identify if binder characterization is limited to functional assays such as ELISA since avidity can compensate for affinity loss. Soluble aggregates negatively affect all those applications, such as for example super-resolution microscopy techniques that rely on the small dimension of antibody fragments to maximize their performances. Therefore, sample monodispersity should be evaluated accurately with biophysical methods [2] and particular attention must be paid to the redox conditions of the cell compartment chosen for protein accumulation during recombinant expression and the optimization of storage buffers. In Fig. 3, the gel filtration profile of a nanobody sample that shows multiple peaks is reported. The first peak corresponds to the void volume and contains mainly aggregates, whereas the second peak corresponds to the monomeric protein. In other words, polymers of different complexity are present in the initial sample, and it is necessary to distinguish and separate these. Of course, this would not be possible by simple SDS-PAGE since apparently, the same target protein is present in all the elution fractions.

#### Soluble fragment of the lymphocyte receptor LLT1

**Fig. 4:**
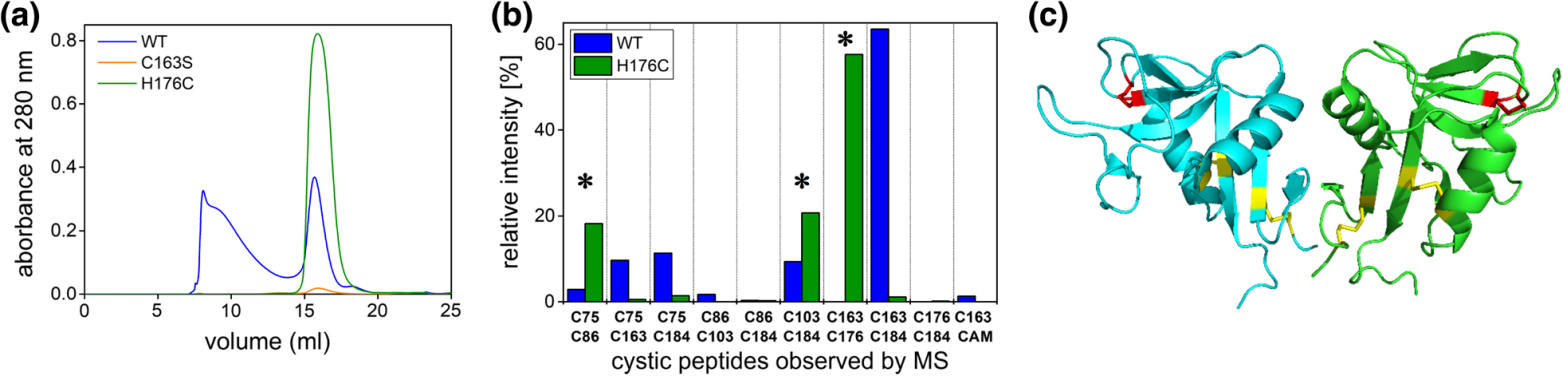
Reconstruction of the disulfide bridge improves the folding and yield of soluble LLT1. **a** Gel filtration of wild-type recombinant soluble LLT1 (blue) and its C163S (orange) and H176C (green) mutants produced in HEK293T cell line. **b** Mass spectrometry analysis of disulfide bond pattern in wild-type LLT1 and its H176C mutant using samples from the peak at 16 ml position on SEC run shown in **a** corresponding to the LLT1 non-covalent dimer. The relative intensity of observed cystic peptides is shown. While in the H176C mutant both the expected two native disulfide bridges (Cys75-Cys86 and Cys103-Cys184) and the third reconstituted bond Cys163-Cys176 were formed (marked by asterisks), in the wild-type LLT1 the odd Cys163 residue paired randomly with other cysteines, leading to protein misfolding and aggregation. **c** Crystal structure of LLT1 (PDB 4QKI) non-covalent dimer (cyan and green) confirmed the expected disulfide bond pattern (in yellow with the reconstituted Cys163-Cys176 disulfide highlighted in red). (Original figure from Vaněk’s lab)

Stable and biologically active protein requiring disulfide bonds for reaching its native fold can be challenging to obtain recombinantly but biophysical analyses can help optimizing the process, as illustrated by the preparation of a soluble fragment of the lymphocyte receptor LLT1 [14]. The gel filtration profile of the wild-type LLT1 construct showed a broad peak corresponding to aggregated material in addition to a well-resolved peak corresponding to the expected non-covalent dimer of LLT1 (Fig. 4A). However, analysis of the disulfide bond pattern by mass spectrometry clearly showed misfolding due to the presence of an unpaired cysteine residue even in the well-resolved peak (Fig. 4B). Therefore, two LLT1 mutants were designed, one in which that particular cysteine residue was removed (leading to a minimal yield, Fig. 4A) and a second one in which another cysteine residue was introduced in order to reconstitute a cystic pair that is conserved in related members of this receptor family. This second mutant was stable, productive, was crystallized, and its 3D structure confirmed the presence of a correctly folded protein containing the reconstituted disulfide bond (Fig. 4C) [15].

#### Prone-to-aggregation proteins

Many proteins we want to purify are completely insoluble, partially soluble, or aggregate for different reasons (Table 1). This condition can be alleviated at the expression level [16] but also poses a significant challenge during downstream purification steps requiring ad hoc strategies [17, 18].

Aggregation can be typically induced by the nucleation of a few proteins that form small and soluble aggregates, which serve as nucleation foci for the subsequent growth of larger insoluble aggregates. The nucleation-growth process can increase with time, temperature, protein concentration, or organic and inorganic contaminants. Notably, an extended lag phase can precede the abrupt formation of large insoluble aggregates [19].

Aggregation of recombinant proteins can start already during expression and may produce both inclusion bodies (IB) and soluble aggregates. Several options have been proposed to limit this problem [16–21]. These include the screening of different bacterial strains, decreasing culture temperature, using modified culture media or different solubility-enhancing fusion proteins such as maltose-binding protein, alternative expression systems such as baculovirus-mediated expression in insect cells, mammalian or cell-free expression, constructs with either amino- or carboxyl-terminal deletions, expressing homologs of the protein of interest, removing flexible loops or residues that affect solubility, and refolding of denatured proteins.

The quality of the overexpressed product must be evaluated by monitoring the ratio between monomeric and aggregated protein step-by-step in small-scale expression and purification tests coupled to SDS-PAGE and analytical gel filtration [22]. Conditions must be determined that lead to a minimal presence of aggregates (both soluble and insoluble) and maximal yield of the native target protein with the correct oligomeric conformation [17, 18].

**Fig. 5:**
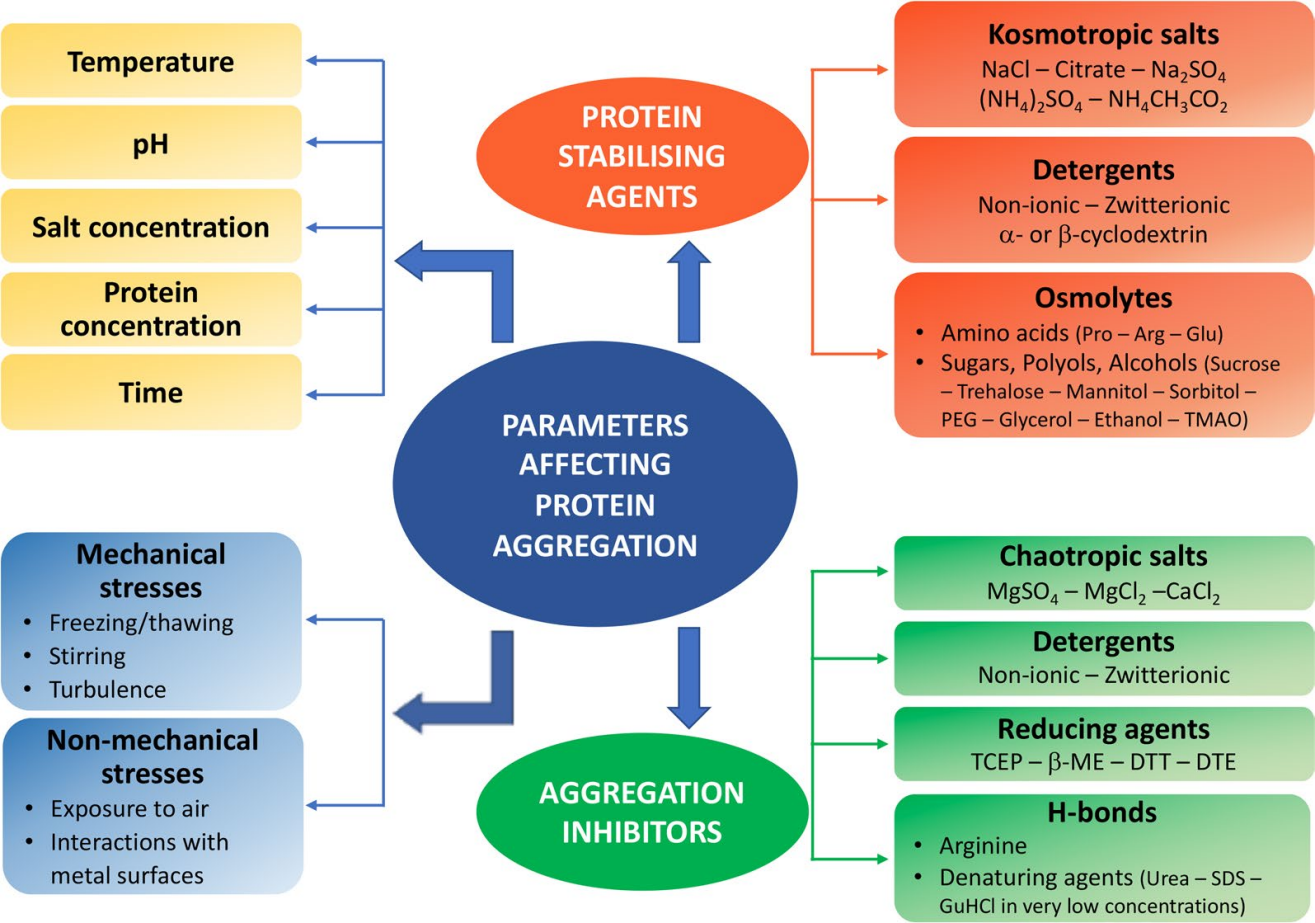
Strategies to alleviate protein aggregation. The factors leading to protein aggregation during purification can be mitigated. Operational time should be reduced as much as possible and samples should be kept at low temperatures throughout the entire purification process. Oxidating conditions, high protein concentrations and destabilizing pH values and salt concentrations should be avoided. Mechanical stress needs to be limited and, when necessary, stabilizing molecules should be added. Some proteins may also have specific requirements for reducing agents and detergents

Although protein solubility during expression is an essential prerequisite before purification, this does not prevent aggregation problems from arising at later stages of the protein production process. In general, factors such as buffer composition, pH value, kosmotropes, or chaotropes should be screened (see Fig. 5). Moreover, the procedures require rapid manipulation at 4 °C, as well as avoiding protein overcrowding during purification steps. The classical strategy of affinity chromatography (e.g., IMAC) followed by protease cleavage to remove the fusion tag and reverse affinity chromatography and size-exclusion chromatography (SEC) [19] could be replaced by affinity chromatography followed immediately by SEC to quickly eliminate soluble aggregates that can serve as aggregation nuclei (see Additional file 1: Example S6). Emphasis should be put on designing a “rapid strategy of purification” for producing and storing the target protein as fast as possible. To achieve this goal, optimizing each purification step and storage conditions should be performed prior to production scale-up.

Although recommended additive concentrations can be found in the literature [17, 20], the optimal range for each protein is highly specific, and the buffer conditions must be fine-tuned for each project. Moreover, there could be a synergistic effect between some of these agents that could prevent different aggregation mechanisms (Additional file 1: Examples S6-S8).

Several experimental methods are routinely used to determine the protein quality and the most suitable buffers, such as visual observation of turbidity, SEC, circular dichroism (CD), light scattering (LS), differential scanning fluorimetry (DSF) and fluorescence-based thermal shift (thermofluor) assays [2]. Screening a pool of additives covering most aggregation mechanisms can considerably reduce cost and efforts [16–18].

#### Crystallization of human CLK1 kinase (how to obtain reproducible batches)

Cdc-like kinase 1 (CLK1) is a dual-specificity kinase capable of autophosphorylation on tyrosine residues and Ser/Thr phosphorylation of its substrates. Its three-dimensional X-ray structure was solved in a previous communication [23], but the procedure was difficult to reproduce. This is not uncommon when working with kinases since their preparation for structural studies can result in non-specific or partial phosphorylation(s) leading to heterogeneous protein populations. Consequently, the recovery of homogeneous preparations of CLK1 without heterogeneous phosphorylation is instrumental to achieve reproducible results in crystallization and co-crystallization experiments with potential inhibitors [24]. First, CLK1 was co-expressed with λ-phosphatase in the same *E. coli* host strain to cleave all possible phosphates from the phosphorylation sites, despite the fact that this approach significantly decreased the final CLK1 yield. His-tagged CLK1 was initially purified by IMAC, after which the eluate was immediately supplemented with 50 mM arginine, 50 mM glutamate and 10 mM DTT to avoid aggregation [25], concentrated and incubated overnight with TEV protease at 4 °C. The correct oligomeric conformation was separated from soluble aggregates by SEC in a buffer containing 5% glycerol and 5 mM β-ME. The last polishing step with anion-exchange chromatography was crucial to separate two different CLK1 populations: the first one, containing a consistent crystallizable non-phosphorylated CLK1 and a second one, consisting of a non-crystallizable partially phosphorylated protein.

It is important to realize that aggregation problems can occur at different stages of the purification process. For example, in the case of CLK1, the change of the cell disruption method to a more aggressive system based on higher pressure and temperature resulted in an unstable CLK1 protein with a higher tendency to aggregate, which was also unsuitable for crystallization assays.

#### Production of stable, ligand-binding-competent Galectin-1

Galectins, a family of carbohydrate-binding proteins with affinity for β-galactosides, are characterized by the presence of a highly conserved carbohydrate recognition domain (CRD) and a shared consensus of amino acid sequences. Galectin-1 is a prototypical lectin with an affinity for β-galactosides that plays a vital role in numerous physiological and pathological processes. It exists physiologically as a non-covalent homodimer, spontaneously dissociating into monomers at a low micromolar range. The carbohydrate ligand-binding (lectin) activity of Galectin-1 depends on three factors: (1) the correct folding and conformation of the carbohydrate recognition domain; (2) the maintenance of the reduced state of the cysteine residues either by addition of thiol-reducing agents or mutation of specific or all cysteine residues present; and (3) the preservation of the ligand-binding site in its free, unbound state.

Reports in the literature describing Galectin-1 recombinant production are commonly based on protocols that do not consider maintaining its lectin activity properly, even in studies where carbohydrate recognition is essential. It is common to find reports where there is no selection of properly folded proteins during purification, where changes in redox state compromise the lectin activity of Galectin-1, or even to find rather incomplete descriptions of how remnants from the purification are removed (i.e., reducing agents or lactose from affinity chromatography).

**Fig. 6:**
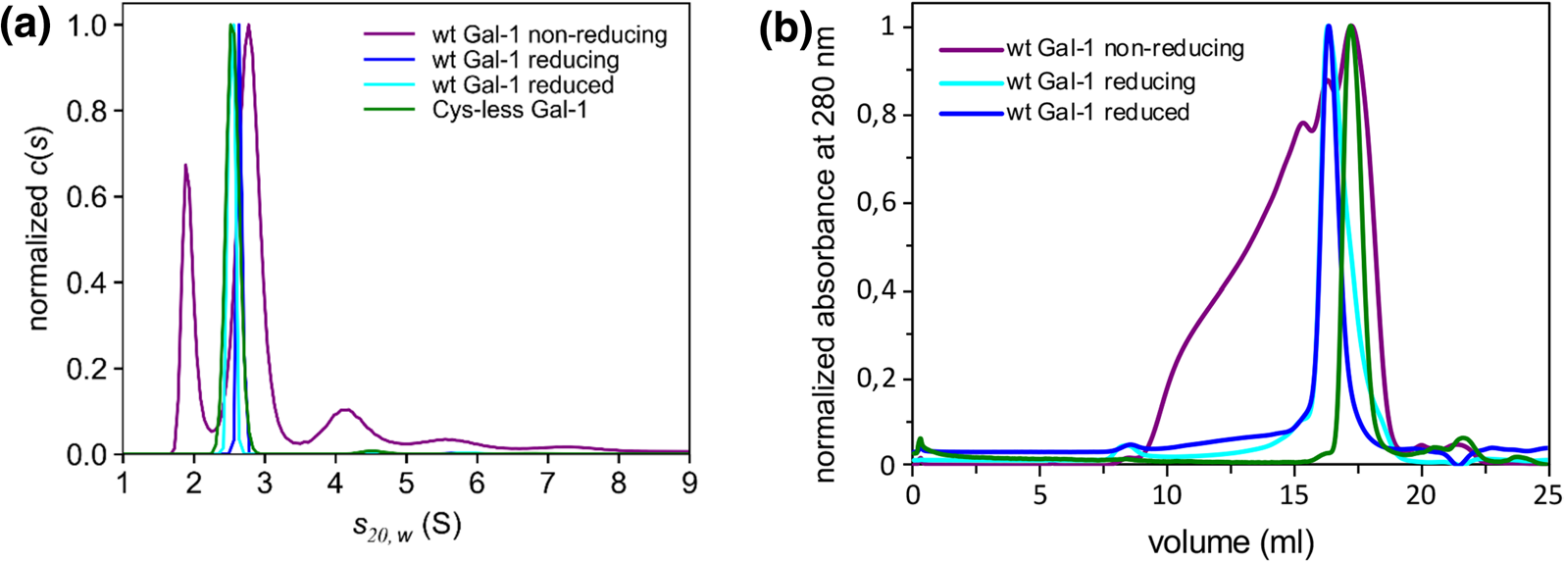
Recombinant Galectin-1 hydrodynamic characterization discloses its instability. **a** Analytical ultracentrifugation shows that wild-type Galectin-1 purified in non-reducing conditions sediments as a mixture of monomer, dimer, and various disulfide-crosslinked oligomeric species (purple curve). In contrast, purification in reducing conditions yields the expected non-covalent dimer (blue curve), which can also be revived from the oxidized sample by transferring it to the reducing conditions (cyan curve). However, the cysteine-less mutant of Galectin-1 is perfectly stable in non-reducing conditions and sediments solely as the expected non-covalent dimer. **b** SEC analysis of wild-type and cysteine-less Galectin-1 purified in non-reducing and reducing conditions complementing the AUC data. Apart from being freshly reduced, wild-type Galectin-1 is not monodisperse, even when purified in reducing conditions where self-oxidation still slowly occurs upon storage. On the contrary, the cysteine-less mutant is perfectly monodisperse in non-reducing conditions. (Original figure from Vaněk’s lab)

In order to study protein–protein interactions of Galectin-1, four different constructs were prepared—tag-less and His-tagged wild-type Galectin-1, and tag-less and His-tagged cysteine-less mutant Galectin-1 (described previously in [26]). The constructs were expressed overnight at 20 °C in the *E. coli* T7 Express strain followed by cell lysis using sonication in the presence of protease inhibitors and centrifugation. The His-tagged constructs were purified by IMAC and eluted with imidazole, whereas the tag-less constructs were purified by affinity chromatography over a lactose-Sepharose column and eluted with lactose. The lack of selection of properly folded proteins, as is the case in IMAC where selection occurs through a His-tag, resulted in protein aggregation; therefore, it is critical to purify Galectin-1 based on its lectin activity to ensure fully functional folded protein preparation. One drawback of this purification method is the presence of lactose in the binding site, which hinders further ligand binding studies where the CRD is involved. Therefore, the next step in the preparation of active, fully binding-competent Galectin-1 was the removal of lactose, where two methods were employed—gel filtration or extensive dialysis. The former proved not efficient enough; only thorough, extensive dialysis against HEPES buffer led to fully restored binding capacity. We can thus conclude that it is superior to purify Galectin-1 over a lactose-based column and remove lactose through extensive dialysis. A different problem arose with the prolonged preparation of the protein—in the absence of any reducing agent, the wild-type Galectin-1 construct completely oxidized following purification, and even in its presence, the protein remained reduced for a short time only (Fig. 6). Knowing that the preparation of recombinant Galectin-1 was in our particular case intended for ligand binding studies with proteins containing disulfide bonds, the presence of reducing agents, even at a lower concentration, could not be considered to avoid destabilizing the binding partner. It became evident that the best candidate for a folded, functional, and stable protein was the cysteine-less mutant of Galectin-1 purified by affinity chromatography over a lactose-based column and dialyzed for removal of lactose. Quality controls were performed by SDS-PAGE in reducing and non-reducing conditions, DLS, nanoDSF, AUC, label-free MST, and ITC, where the redox conditions, protein dispersity, thermal stability, and lectin activity were thoroughly assessed (Abreu et al., manuscript in preparation). In all instances, the cysteine-less mutant of Galectin-1 performed equally to wild-type Galectin-1 but with enhanced long-term stability, thus proving to be a valuable candidate for ligand binding studies where Galectin-1 ought to be correctly folded and active.

#### Endotoxin removal

Endotoxin contamination has been the bane of the bioprocessing industry since its inception. Endotoxins are everywhere: They are toxic and/or interfere with every type of therapeutic, diagnostic, and research product; they are indestructible within the limits of product tolerance and they are difficult to remove [27–29]. Proteins produced in *E. coli* are contaminated by endotoxins, which are lipopolysaccharides (LPS) of the outer cell membrane of Gram-negative bacteria. LPS binds to and activates toll-like receptors (TLR), thereby inducing the secretion of pro-inflammatory cytokines and systemic inflammation [30]. High LPS blood concentrations, namely above 0.6 endotoxin activity units/ml, are associated with a high risk of severe sepsis, septic shock, and mortality in humans [31]. Thus, endotoxin removal is fundamental when proteins are used for animal and human studies or for several in vitro analyses using cells in culture (Table 1).

Endotoxin removal methods have been based on positively charged chromatography (anion exchange) and affinity chromatography using poly-cation ligands such as poly-L-lysine (PLL) or immobilized polyamine (polymyxin B). Alternatively, the addition of surfactant Triton X-114 exploits the hydrophobicity of the endotoxin lipid A moiety [32]. The protocol involves an incubation step at room temperature in the presence of the surfactant to promote association with lipid A, followed by refrigeration to cause the detergent to gelatinize, facilitating its removal with still-associated endotoxin. The protocol can be adapted for simultaneous metal chelate affinity purification and endotoxins clearance: once the protein target is bound to IMAC (Ni) resin, it is washed with 50 column volumes of buffer containing 0.1% (v/v) of Triton X-114 followed by 20 column volumes of the buffer without detergent at 4 °C before imidazole elution [33]. One percent (v/v) of Triton X-114 is used for IB washing before solubilization [34]. A preliminary ion exchange step is beneficial as LPS is highly negatively charged and strongly binds to anion exchange columns (at pH 8.0, it should elute around 500 mM NaCl), whereas the protein of interest is recovered in the flow-through.

To avoid endotoxin contaminations originating from prior *E. coli* purifications on the same chromatography workstation, it is opportune to incubate the chromatographic pump/fluidic system overnight in 0.5 M NaOH or 4 h in 1.0 M NaOH and flush all the valves and lines before starting the purification process. Ideally, buffers should be prepared in LPS-free water (or in tap water, which usually is LPS-free when collected after 4–5 min of tap purge). Be aware that many commercial salt stocks also contain LPS; therefore, LPS-free (cell culture-grade) compounds should be purchased for these applications.

For the purification, use new columns or columns that have only been used in other LPS-free purifications. Some resin material contains endotoxin as well (e.g., StrepTactin, as it is produced in *E. coli*), so this needs to be washed carefully beforehand, too. A *Limulus amoebocyte* lysate (LAL) assay is required to assess the final amount of LPS in samples and verify that it is below the limit required for the specific applications.

#### Purification of protein “X”

Protein “X” from *M. tuberculosis* was expressed in *E. coli* BL21(DE3) Star as a His_6_-TrxA fusion protein and purified from the soluble fraction through immobilized-metal affinity chromatography (IMAC). The TrxA-tag was cleaved off using the TEV protease and the target protein was recovered by an additional IMAC step (Additional file 1: Fig. 2A). The endotoxin removal was carried out using a spin column (Column 1: ThermoFisher, cat. #88,277) containing a resin made of poly-ε-lysine covalently attached to porous cellulose beads following the standard manufacturer procedures. However, this standard protocol was not successful in bringing the endotoxin concentration in the protein sample to the set threshold (< 10 EU/ml; < 0.01 EU/μg) (Additional file 1: Fig. 2B). Hence, to have a final product suitable for mouse immunization, several approaches were tested to abate endotoxin level significantly. Firstly, an improvement of the “Column 1” protocol was explored. For Protein “X”, the optimized conditions included a longer incubation time of 2–3 h (up to overnight) at room temperature on a rotating mixer, a sample recovery step from the spin column at 500 × g for 3 min, and the usage of Tris–HCl as elution buffer. With this method, the final endotoxin concentration was around 8 EU/ml, namely tenfold lower than the one obtained in standard conditions. Alternatively, additional chromatographic steps such as anion exchange chromatography (AIEX) and SEC were introduced considering Protein “X” properties such as isoelectric point (9.36) and molecular weight (29.10 kDa). The AIEX polishing step was performed using buffers at pH 8.0, thus allowing the negatively charged lipopolysaccharide (endotoxin) molecules to bind to the resin and collect the unbound Protein “X” in the flow-through and/or during washing. As a result, the endotoxin concentration ranged between 20–30 EU/ml with a protein recovery of 80% from the total protein present in the preceding step (2^nd^ IMAC). The third method exploited size-exclusion chromatography and led to a final endotoxin concentration < 10 EU/ml. However, a considerable sample loss was observed (protein recovery: 20%), probably due to protein precipitation or unspecific binding to the column. Both AIEX and SEC can be combined to an endotoxin removal process with spin Column 1, thus having a final product that reaches an acceptable endotoxin level. Importantly, to avoid any external endotoxin contaminations [35], all the solutions were freshly made using endotoxin-free water. The FPLC system was washed thoroughly with 1 M NaOH after every purification process, and when possible, sample handling was done under a sterile working environment. The most effective strategies that led to the best endotoxin removal with minor sample loss were the usage of spin Column 1 (optimized conditions, O.C.) and the combination of AIEX with spin Column 1 (standard condition, S.C.), where the endotoxin concentration is reduced to level < 0.01 EU/μg and the protein recovery is > 70% and > 50%, respectively.

#### Protein complexes

The expression and purification of protein complexes can be challenging, and the optimal conditions will vary on a case-to-case basis (Table 1). In some cases, the individual subunits can be expressed separately and then assembled into a protein complex in vitro. However, individual subunits are often not stable or unable to fold into their native 3D structure in the absence of their interaction partners. In these particular cases, simultaneous co-expression of individual subunits is required to form functional protein complexes. Fortunately, over the years, various heterologous multiprotein expression tools have been developed for standard host organisms such as *E. coli* [36, 37], insect [38–40], and mammalian cells [41]. Some methods rely on co-transformation, co-infection, or co-transfection, whereas others allow simultaneous expression of various complex subunits from a single plasmid or virus. For example, an exciting strategy developed to produce protein complexes in insect cells relies on the expression of a single polypeptide chain containing the TEV protease followed by all complex subunits, each of them (including the TEV) being separated by the TEV cleavage site. This approach has the advantages of expressing all subunits under the same promoter and having them in close proximity for correct complex assembly [42, 43].

Construct design is of utmost importance for recombinant expression of multiprotein complexes, as care needs to be taken that tags introduced for purification and/or detection purposes do not hinder proper complex assembly in the cells. When little information is available about the exact complex composition, stoichiometry, and structural arrangement of the separate subunits, this can take several rounds of optimization. It is essential to carefully assess complex integrity and stability throughout the various steps of the purification workflow. Many different methods can be used for this, the simplest of which is SDS-PAGE visualization of the individual subunits. Homogeneity can be evaluated by SEC, while SEC coupled to multi-angle light scattering (SEC-MALS) offers the additional benefit of also being able to assess the molar mass. Additionally, mass photometry [44] or native mass spectrometry [45, 46] can be used as well to check the masses of the entities present in the purified sample. When using methods such as SEC, SEC-MALS, or mass photometry, it is crucial to keep in mind that disassembly of the complex into various subunits can be observed when working at concentrations below the *K*_D_ of the individual interactions. If complex disassembly occurs during certain steps of the purification workflow, an adaptation of the chromatographic methods and/or the purification buffers might be necessary. For buffer optimization, techniques such as thermofluor or differential scanning fluorimetry (DSF) can be very helpful [47]. In some cases, limiting the number of steps in the chromatographic workflow might also be more effective to avoid losing some of the (more weakly interacting) subunits by “over-purification”.

#### Antigen–antibody complex purification

An antibody bound to its antigen represents a specific example of a protein complex. Antibody-antigen complexes are often co-crystallized to study the interaction modalities and then such structural information can be used for engineering the antibody into a variant with improved binding features. The increasing availability of small antibody fragments has facilitated both the structural characterization of protein complexes and the modification of recombinant binders. Conventionally, the antigen and antibody are purified separately and then mixed together to form a stoichiometric protein complex. However, it has been demonstrated that they can also be co-expressed and co-purified [48], which implies only a single purification. Furthermore, co-expression can be exploited to stabilize labile antigens by means of antibody binding and also allows the expression of one of the partners without any tag. In this particular case, SDS-PAGE and gel filtration are generally sufficient to evaluate the purification procedure and the quality of the protein complex.

## Conclusions

**Fig. 7:**
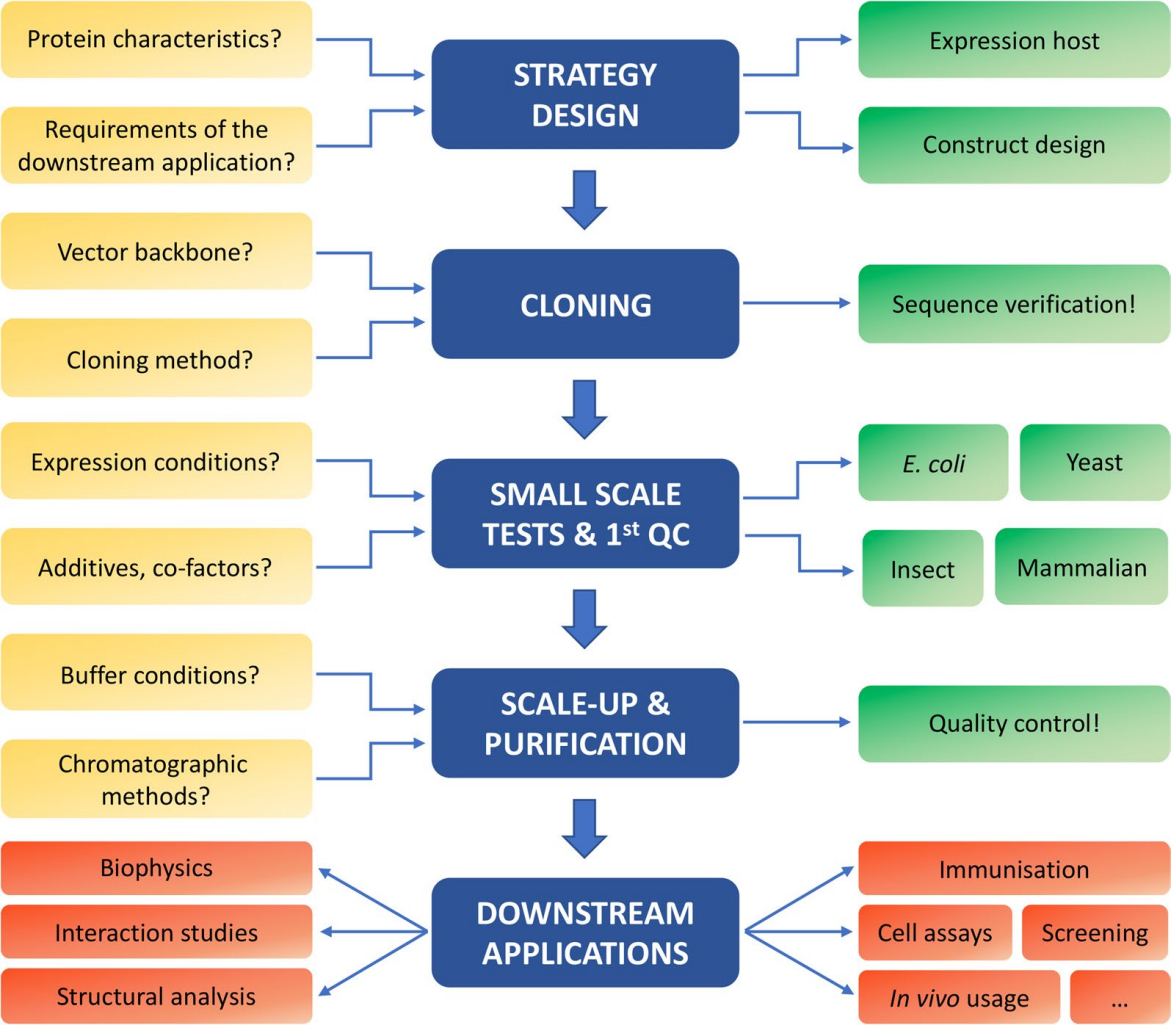
Workflow of a protein production process. Each protein production process starts with the strategy design. The biochemical characteristics of the protein of interest and the intended downstream applications, as indicated in Table 1 and Fig. 1, need to be considered when deciding which expression host organism to use and how to design the expression construct. After cloning the gene(s) of interest into a suitable expression plasmid, the sequence must be verified. Next, the most optimal expression conditions in the host organism of choice (usually *E. coli*, yeast, insect, or mammalian cells) are determined. This includes the screening of various parameters such as the expression strain, the expression medium, growth temperature, time, etc. Once the best condition to obtain soluble protein(s) has been found, one can proceed to large-scale protein purification. At this step, it is important to decide on the chromatographic methods that will be used (affinity chromatography, ion exchange, size-exclusion chromatography, …) and to find buffer conditions in which the protein remains in a soluble, properly folded state. Appropriate quality controls throughout the entire process are important to make sure the protein of interest is stable, non-aggregated (Fig. 5), and in a native state. The purified protein can then be used in various downstream applications (Table 1), such as biophysical characterization, interaction studies, structural analysis, immunization, cell assays, etc.

Purified protein reagents are used in a large variety of scientific fields. Many reviews exist for protein production on an academic scale, detailing popular strategies (for examples, see references [49, 50]). These strategies represent good starting points for producing pure, soluble proteins in close-to-milligram quantities and are implemented on a daily basis by professionals in research groups and core facilities with strong expertise in (recombinant) protein expression, purification, and standard characterization techniques. However, these are general indications that might require specific adaptations when downstream applications require particular conditions, such as endotoxin-free proteins for animal experiments or nuclease-free proteins for nucleic acid-interaction studies (see Fig. 1 and Table 1). Similarly, certain proteins possess intrinsic biochemical features that need to be considered when designing the expression and purification strategies, such as proteins that are prone to aggregation, possess disulfide bonds, or have a high affinity for nucleic acids. In this work, we presented experimental cases describing ad hoc strategies for producing such types of proteins. In these specific cases, a proper strategy needs to be designed upfront, starting from the choice of the expression system, design of the constructs (choosing appropriate tags and domain boundaries when necessary) and development of the protein purification workflow, including quality controls at various steps (Fig. 7). Some specific analyses not included in the initial set of guidelines for protein quality control assessment [2] might become essential in such specific experimental contexts. This proactive process of strategy design is comparable to Quality Assurance (QA) in pharmacological and biotechnological industries. QA is defined as the ensemble of activities required to ensure good quality in the processes used for product development and includes many methods, all aiming at preventing defects in the final product. With the introduction of quality control guidelines [1, 2] and by generating a better understanding of the specific requirements for proteins to be used as reagents in biological applications, we hope to develop proper standards for purifying high-quality proteins in a broad range of academic labs as well.

## Supplementary Information

**Additional file 1.** Supplementary Materials

## Data Availability

Not applicable.

## Abbreviations

AUC: Analytical Ultracentrifugation
β-ME: Beta-mercaptoethanol
CD: Circular Dichroism
CE: Capillary Electrophoresis
Cryo-EM: Cryo-Electron Microscopy
DLS: Dynamic Light Scattering
DTNB: 5,5'-Dithio-bis-(2-nitrobenzoic acid)
DTT: Dithiothreitol
ELISA: Enzyme-Linked ImmunoSorbent Assay
EMSA: Electrophoretic Mobility Shift Assay
FPLC: Fast Protein Liquid Chromatography
IB: Inclusion Body
IgG: Immunoglobulin G
IMAC: Immobilised Metal Affinity Chromatography
IP: Immunoprecipitation
ITC: Isothermal Titration Calorimetry
LPS: Lipopolysaccharides
MS: Mass Spectrometry
MST: MicroScale Thermophoresis
Nano-DSF: Nano-Differential Scanning Fluorimetry
NMR: Nuclear Magnetic Resonance
QC: Quality Control
SDS-PAGE: Sodium Dodecyl Sulfate-PolyAcrylamide Gel Electrophoresis
SEC: Size Exclusion Chromatography
SEC-MALS: Size Exclusion Chromatography coupled to Multi-Angle Light Scattering
TCEP: Tris-(2-carboxyethyl) phosphine hydrochloride
TEV protease: Tobacco Etch Virus protease
