# Supplementary material for: Protein purification strategies must consider downstream applications and individual biological characteristics
Source: Microb Cell Fact. 2022 Apr 7;21:52. doi: 10.1186/s12934-022-01778-5 (PMC8991485; doi:10.1186/s12934-022-01778-5)
Supplement: Supplementary file 1 — Additional file 1. Supplementary Materials [file 12934_2022_1778_MOESM1_ESM.docx]

**Supplementary Material**

**Supplementary Figures**

**
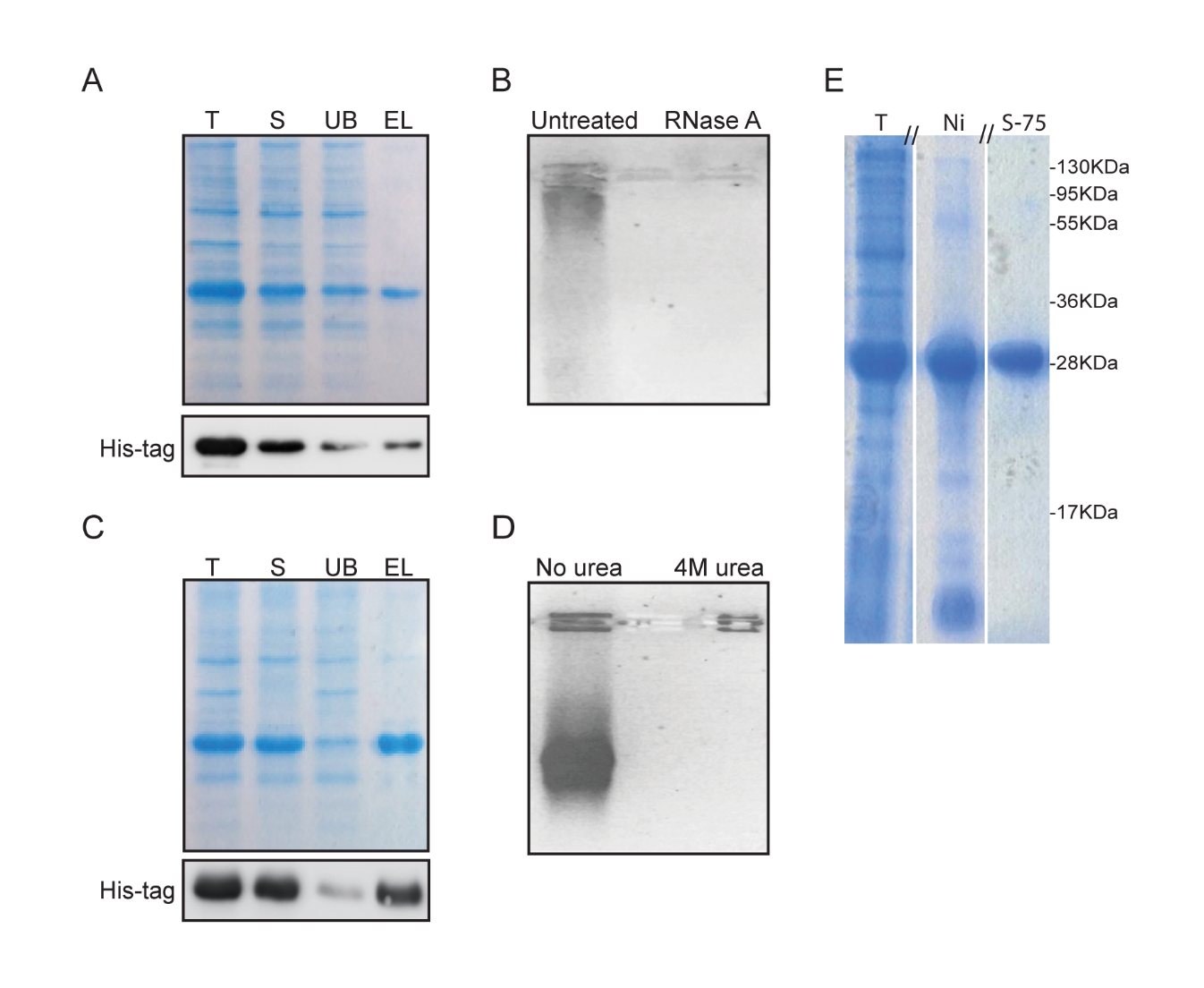
**

Supplementary Figure S1. Purification of dsRBEC

A) dsRBEC was purified on Ni Sepharose under native conditions. Samples of total lysate (T), soluble fraction (S), unbound fraction (UB) and eluate (EL) were resolved by SDS-PAGE. dsRBEC was visualized with Coomassie dye and by western blot analysis using an anti-His antibody. B) Samples of EL electrophoresed on 1 % agarose and stained with ethidium bromide. Treatment with 10 μg/ml RNase A eliminated the nucleotide-dependent staining. C) dsRBEC was purified on Ni Sepharose under denaturing conditions (4 M urea). Samples of total lysate (T), soluble fraction (S), unbound fraction (UB) and eluate (EL) were analyzed as in (A). D) Equal amounts of eluted protein, isolated under native or denaturing conditions, were electrophoresed and stained with ethidium bromide. E) SDS-PAGE analysis of optimized dsRBEC purification (15 % bis-acrylamide gel stained with Coomassie dye): T, total crude lysate before purification; Ni, eluate from 4 ml Ni Sepharose column; S-75, eluate from final purification on Superdex 75 column. The picture was originally published in [1] and PLoS One kindly allowed its reproduction.


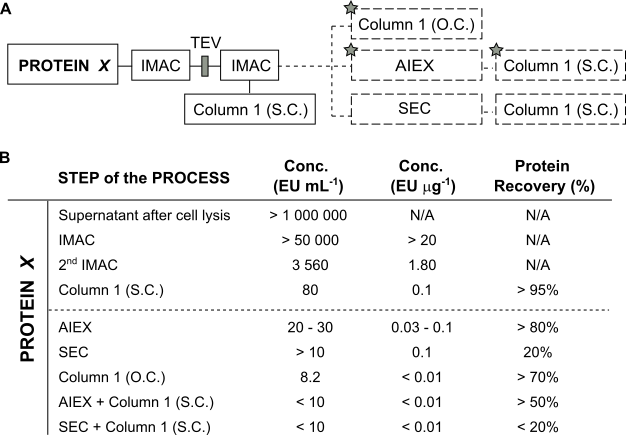


Supplementary Figure S2. Specific features of Protein “X” purification.

A) Process flowchart. In the initial protocol, the fusion protein X underwent the first step of metal affinity purification, a TEV protease digestion to remove the tag and a second IMAC that enabled the recovery of protein X in the flow-through (full lines). This was loaded on poly-lysine column 1 to remove the endotoxins following the manufacturer’s protocol. Three alternatives were tested after the second IMAC step. Either the same poly-lysine column 1 was used with a modified chromatography protocol, or a preliminary step using either ion-exchange or gel filtration chromatography anticipated the final endotoxin stripping on column (dashed lines) 1. B) Endotoxin concentrations and recovery of protein X at the successive purification steps of the different protocols. S.C., standard conditions, O.C., optimized conditions.

**Supplementary Examples**

**Nucleic acid-binding proteins**

S1: Tn5 transposase

Tn5 transposase is an enzyme that can be used to prepare NGS libraries. The protein can be expressed in *E. coli* as a His_6_-Sumo3 fusion protein and purified by a combination of affinity chromatography and size exclusion chromatography [1]. However, without introducing a specific nucleic acid removal step in the purification workflow, one will end up with *E. coli* reads upon the usage of the enzyme for NGS library generation due to contamination of the Tn5 preparation with *E. coli* DNA. To avoid this issue, PEI precipitation of the cleared lysate followed by another centrifugation step was introduced before the first immobilized metal affinity chromatography (IMAC) [1]. In this case, however, care has to be taken that the IMAC material is compatible with the presence of some PEI as, for some commercially available resins, this leads to stripping of the beads. Another possibility would be to perform Heparin chromatography right after the IMAC. However, as the Tn5 transposase is somewhat sensitive to the lower salt concentrations required for efficient binding to the Heparin column, the PEI precipitation would be the preferred method in this specific case.

S2: A chimeric protein consisting of single-chain antibody ScFvJ591 fused to the human dsRNA-dependent protein kinase, PKR

The chimeric protein contains the double-stranded RNA (dsRNA) binding domain of PKR tethered to a single chain anti-PSMA antibody. This protein vector selectively delivers synthetic dsRNA, polyinosinic/polycytidylic acid (polyIC), to prostate tumors by targeting the prostate-specific membrane antigen (PSMA), which is overexpressed on the surface of prostate cancer cells.

Similar to example 1.2 (dsRBD-EGF-Chimera) described in the main text, only the addition of a high concentration of urea (4 M) to the *E. coli* lysis buffer led to the release of the bound contaminant RNA from the chimeric protein and allowed binding to IMAC column (work performed in Lebendiker’s lab, unpublished). After on-column refolding with optimized buffers, the protein underwent a final polishing step on a highly resolutive cation exchange column Fractogel^®^ EMD SO3- (33 × 1.6 cm ~ 66 ml column) with a gentle NaCl gradient in order to get rid of partially cleaved protein and aggregates.

When complexed with polyIC, the chimera demonstrates selective and efficient killing of prostate cancer cells. The treatment causes the targeted cancer cells to undergo apoptosis and secrete toxic cytokines. In a “bystander effect”, these cytokines kill neighboring cancer cells that do not necessarily overexpress PSMA and activate immune cells that enhance the killing effect. The strong effects of the targeted polyIC are demonstrated on both 2D cell cultures and 3D tumor spheroids.

**Proteins used as antigens**

S3: Inclusion bodies provided antigen suitable for the recovery of valuable monoclonal antibodies

Her2 is a very well-studied receptor because its overexpression contributes to tumor growth in several organs. Both conventional and fragment anti-Her2 antibodies have been produced, mostly using the receptor extracellular domain (ECD) as the corresponding antigen. Its complex structure stabilized by multiple disulfide bonds prevents the production of native ECD in bacteria. However, eukaryotic expression of Fc-Her2ECD is expensive and requires fusion tag cleavage and removal, resulting in low yields. Therefore, we (de Marco’s lab, unpublished results) opted for the expression of an ECD fragment in the bacterial cytoplasm aiming at the accumulation of inclusion bodies (IB) to produce the required antigen for mouse immunization. These IB were effectively purified to apparent homogeneity (Supplementary Figure S3), solubilized in urea and used for animal immunization. The immune sera were tested by western blot using an antigen sample produced in HEK cells and specifically recognized a band corresponding to Her2ECD.


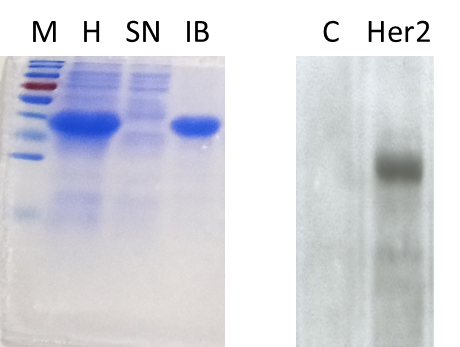


Supplementary Figure S3. Her2-specificity of antibodies obtained by mouse immunization with IB.

The ECD domain of Her2 was highly expressed in the bacterial homogenate (H) but was completely insoluble (no signal in the supernatant fraction SN). Once recovered as a homogeneous IB sample, it was used for animal immunization. The pre-immune serum did not react with purified Her2 (C), whereas the immune serum recognized its antigen (Her2).

S4: Effect of antigen quality on panning output

*In vitro* panning promotes the selection of binders specific to the available epitopes. With respect to native proteins, aggregates can combine into structures that provide new epitopes. Their presence can guide the recombinant antibody selection towards artifacts that do not correspond to any native epitope. Therefore, the panning can be technically successful but fail to provide useful binders. Once we (de Marco’s lab, unpublished data) received a protein sample to use as an antigen for panning a nanobody phage display library. The selected clones that were tested positive in ELISA against the antigen sample were further used to detect the native target protein but failed. Repeated ELISA experiments confirmed that the selected nanobodies strongly bound to the protein sample used for panning but not the native protein. A gel filtration analysis of the protein sample showed that most of it was present as aggregates (Supplementary Figure S4). The take-home message was clear: the quality of any reagent must always be tested before its use to avoid downstream failures!


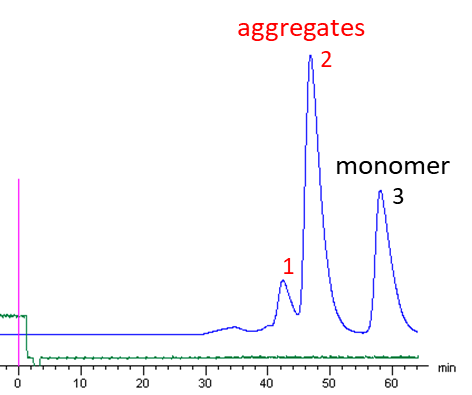


Supplementary Figure S4. Heterogenous antigen composition

The presence of different antigen peaks, as detectable by gel filtration and corresponding to monomeric and aggregated forms, compromised the output of the downstream panning process, favoring the isolation of nanobodies specific for epitopes exclusively present in the antigen aggregates (peaks 1 and 2).

**Proteins that bind to divalent cations or other co-factors**

S5: Zinc binding on structured and disordered regions in the multidomain STIL protein

The binding of metal ions is an important regulatory mechanism in proteins. In the SCL-interrupting locus protein (STIL), which plays an essential role in cell proliferation and survival, Zn^2+^ binding to disordered regions commonly induces a disorder-to-order transition and leads to a gain of structure or oligomerization. The binding of Zn^2+^ ions has different effects on structured and disordered domains in the same multidomain protein.

The N-terminal domain of STIL was expressed as an HLT-fusion protein in *E. coli* cells grown at 37°C in 2× YT medium [2]. At an OD_600nm_ of 0.2, a heat-shock treatment was performed by adding 0.1 % glycerol and 0.1 mM potassium glutamate to the cell culture medium and incubating the *E. coli* cultures at 42 °C for 20 min (leading to heat-shock induced chaperone accumulation). Afterward, the temperature was reduced to 37 °C, and the bacteria were grown until an OD_600nm_ of 0.7. Induction was started by adding 0.1 mM ZnCl_2_ and 0.1 mM IPTG and carried out overnight at 22 °C. Both the heat-shock treatment and overnight induction at 22 °C highly reduced protein aggregation. Moreover, it was essential to use buffers with a relatively high NaCl concentration (0.3 M) and glycerol (2-5 %) during the purification procedure to reduce aggregation. 5 mM β-ME was also added in order to reduce free cysteines. The protein was purified by IMAC and SEC, and the presence of zinc in the final protein sample was corroborated by atomic absorption spectrophotometry.

**Prone-to-aggregation proteins**

S6: Development of a new protocol for the expression and purification of human iASPP Pro

In order to perform protein:protein interaction studies with the human iASPP Pro, an intrinsically disordered protein (IDP) with a high tendency to aggregation, the expression and purification protocol had to be optimized to tackle efficient production of this prone-to-aggregation protein [3]. HLT-iASPP Pro was expressed in *E. coli* Rosetta 2 (Novagen) using the HLT-tag consisting of the lipoyl domain fusion tag with an N-terminal His-tag and an optimized Tobacco Etch Virus (TEV) protease cleavage site in order to increase solubility [4]. Bacteria were grown in 2× YT medium containing 1 % glucose at 37 °C. Induction was performed at A_600nm_ of 0.6 with 0.4 mM IPTG. Cells were harvested after 16 h of incubation at 17 °C. Buffer optimization showed that Tween 20 is required for the protein to remain in solution. Therefore, 1 % Tween 20 was used during cell lysis and subsequently reduced to 0.001 % during the protein purification process and storage. HLT- iASPP Pro was purified using an IMAC column in combination with a buffer at pH 7.0 containing 300 mM NaCl, 10 % glycerol, and 0.001 % Tween 20. The concentration of stabilizing additives added during cell lysis to aid solubilization (Tween 20 in this case) can often be drastically reduced during the last purification steps [4,5]. Once the iASPP Pro was eluted from the IMAC column, it was immediately loaded on a 500 ml preparative SEC column to quickly separate the monomeric species from soluble aggregates. The final protein was stored at -80 °C.

To prepare untagged iASPP Pro, the HLT fusion tag was removed using TEV protease overnight at 4 °C. The His_6_-tagged TEV protease, the HLT fusion-tag, impurities, and any residual uncleaved HLT-iASPP Pro were removed by an additional nickel column step using a Chelating Sepharose^®^ FF 4 ml column. Finally, monomeric iASPP Pro was purified using a preparative SEC column. In our hands, this procedure was more efficient than the classical procedure of doing the TEV protease incubation step immediately after the first Ni column. The reason for this is that the presence of soluble aggregates after the first Ni column accelerates the aggregation process of HLT- iASPP Pro during TEV incubation. Aggregated HLT- iASPP Pro cannot be cleaved by TEV protease and, as a consequence, the final yield of untagged iASPP Pro will be lower.

S7: Production of *Arabidopsis* VIP1 and *Agrobacterium* VirE2

The disordered region of the *Arabidopsis* VIP1 binds the *Agrobacterium* VirE2 protein outside its DNA-binding site [6]. Both VIP1 and VirE2 are difficult to express and purify due to their tendency to aggregate. In order to obtain both proteins in their correct oligomeric conformation, both proteins were expressed using the same solubility-enhancing HLT-tag consisting of the lipoyl domain fusion tag with an N-terminal His-tag and an optimized Tobacco Etch Virus (TEV) protease cleavage site [3]. Transformed bacteria were subjected to a 20 min heat shock at 42 °C after the addition of 0.1 % glycerol and 0.1 mM potassium glutamate (in order to stimulate internal chaperone production), followed by isopropyl-β-D-1-thiogalactopyranoside (IPTG) induction for 16 h at 17 °C [7].

HLT-VIP1 was purified using an IMAC column in combination with a buffer at pH 8.0 containing 500 mM NaCl, 10 % glycerol, and 1 M urea. Once eluted from the IMAC column, the HLT-VIP1 was immediately loaded on a 500 ml preparative SEC column in similar buffer conditions but without urea to quickly separate the monomeric species from soluble aggregates. The final protein was stored at -80 °C. The urea added during cell lysis aided solubilization, and the concentration could be drastically reduced or eliminated during the last purification steps [4,5]. The HLT-VirE2 was purified similarly using the same buffers but without the need for adding urea during cell lysis to enhance solubilization.

S8: Optimized purification of the hepatitis B virus core protein assembly domain

The hepatitis B virus core protein (Cp, 21 kDa) is one of the most promising targets for antiviral drug development. It comprises 183-185 amino acids (genotype dependent) and can be sectioned into two domains: the N-terminal assembly domain (149 aa, 17 kDa), which is alpha-helix rich, and the C-terminal RNA-binding domain (34-36 aa, 4 kDa) [8]. The assembly domain can be expressed in *E. coli* without RNA-binding domain as Cp dimers, which spontaneously aggregate to form capsids. This self-assembly can be influenced by protein concentration, temperature, and ionic strength [9,10].

Although the purification of the HBV Cp dimers was already performed and described in detail by Zlotnick *et al*. [10], we (work performed in May’s lab, unpublished) chose to optimize the production process because, in our hands, only low yields (< 1 mg protein out of 4 l *E. coli* expression culture) were obtained after a long purification set-up using four size exclusion columns (SEC) and three concentration steps. Following the original protocol, the Cp assembly domain was expressed with an additional cysteine as Cp150, with all native cysteines replaced by alanines [3]. After expression in *E. coli* Rosetta 2(DE3) cells (Novagen), the cells were lysed with the commercial Qproteome Bacterial Protein Prep Kit (QIAGEN # 37900) instead of sonicated without an observed decrease in activity later. After ammonium sulfate precipitation at 40 % saturation, the assembled Cp150 was purified in a first step using the mixed-mode column HiTrap CaptoCore 700, 1 ml, combining size exclusion with affinity. By replacing the first SEC from the original protocol (Sepharose CL-4B column, 350 ml) with a mixed-mode column, we reduced the time necessary for this step and avoided sample dilution typical for SEC columns. Therefore, no concentration step via Amicon Stirred Cell (Millipore) was necessary, reducing protein loss due to membrane binding. The flow-through of the CaptoCore 700 containing the capsids was dialyzed into the SEC buffer containing 50 mM sodium bicarbonate and 5 mM DTT at pH 9.6. Afterward, the capsids were disassembled using 3 M urea. The resultant dimers were separated on Sephacryl S-300 HR XK 50/60 SEC column, and the dimer containing fractions were combined, concentrated up to 2 mg/ml, and dialyzed into 50 mM HEPES buffer pH 7.5 containing 5 mM DTT. After the dialysis, the pooled dimers were again reassembled using 0.5 M NaCl. The newly formed capsids were again purified using the CaptoCore 700 and afterward disassembled into dimers and purified as described above. The repeated assembly and disassembly were crucial to purify only active, assembly-competent dimers. If these steps were omitted, a significant drop in assembly activity of the Cp150 dimers was observed. In addition, it is important that all steps during the purification process are performed at 4 °C. The final Cp150 dimers were concentrated and stored at –80 °C for several months. The average yield was 29 mg out of 1 l of *E. coli* expression culture. A short summary of the optimized protocol was already mentioned by Corcuera *et al*. [8].

**References**

[1] Edinger N, Lebendiker M, Klein S, Zigler M, Langut Y, Levitzki A. Targeting polyIC to EGFR over-expressing cells using a dsRNA binding protein domain tethered to EGF. PLoS One 2016;11(9):e0162321.

[2] Hennig BP, Velten L, Racke I, Tu CS, Thoms M, Rybin V, Besir H, Remans K, Steinmetz LM. Large-Scale Low-Cost NGS Library Preparation Using a Robust Tn5 Purification and Tagmentation Protocol. G3 (Bethesda) 2018;8(1):79-89. doi: 10.1534/g3.117.300257

[3] Amartely H, David A, Shamir M, Lebendiker M, Izraeli S, Friedler A. Differential effects of Zinc binding on structured and disordered regions in the multidomain STIL protein. Chem Sci 2016;7(7):4140-7. doi: 10.1039/c6sc00115g

[4] Iosub Amir A, van Rosmalen M, Mayer G, Lebendiker M, Danieli T, Friedler A. Highly homologous proteins exert opposite biological activities by using different interaction interfaces. Sci Rep 2015;5:11629. doi: 10.1038/srep 11629

[5] Lebendiker M, Danieli T. Production of prone to aggregate proteins. FEBS Lett 2014;588(2):236-246. doi: 10.1016/j.febslet.2013.10.044

[6] Leibly DJ, Nguyen TN, Kao LT, Hewitt SN, Barrett LK, Van Voorhis WC. Stabilizing additives added during cell lysis aid in the solubilization of recombinant proteins. PLoS One 2012;7(12):e52482. doi: 10.1371/journal.pone.0052482

[7] Maes M, Amit E, Danieli T, Lebendiker M, Loyter A, Friedler A. The disordered region of *Arabidopsis* VIP1 binds the *Agrobacterium* VirE2 protein outside its DNA binding site. Prot Eng Des Sel 2014;27(11):439-446. doi:10.1093/protein/gzu036

[8] Diamant S, Eliahu N, Rosenthal D, Goloubinoff P. Chemical chaperones regulate molecular chaperones in vitro and in cells under combined salt and heat stresses. J Biol Chem 2001;276(43):39586-39591. doi: 10.1074/jbc.M103081200

[9] Corcuera A, Stolle K, Hillmer S, Seitz S, Lee J-Y, Bartenschlager R, Birkmann A, Urban A. Novel non-heteroarylpyrimidine (HAP) capsid assembly modifiers have a different mode of action from HAPs *in vitro*. Antiviral Res 2018;158:135-142. doi: 10.1016/j.antiviral.2018.07.011

[10] Zlotnick A, Johnson JM, Wingfield PW, Stahl SJ, Endres D. A theoretical model successfully identifies features of hepatitis B virus capsid assembly. Biochemistry1999; 38(44):14644-14652. doi: 10.1021/bi991611a

[11] Zlotnick A, Lee A, Bourne CR, Johnson JF, Domanico PL, Stray SJ. *In vitro* screening for molecules that affect virus capsid assembly (and other protein association reactions). Nat Protoc 2007;2(3):490-498. doi: 10.1038/nprot.2007.60
